# Food Insecurity and Eating Pathology in Adolescents

**DOI:** 10.3390/ijerph18179155

**Published:** 2021-08-30

**Authors:** Brittany H. Kim, Lisa Ranzenhofer, Jill Stadterman, Yvette G. Karvay, Natasha L. Burke

**Affiliations:** 1 New York State Psychiatric Institute, New York, NY 10032, USA; brittany.kim@nyspi.columbia.edu; 2 Department of Psychiatry, Columbia University Irving Medical Center, New York, NY 10032, USA; 3 Department of Psychology, Fordham University, Bronx, NY 10458, USA; jstadterman@fordham.edu (J.S.); ykarvay@fordham.edu (Y.G.K.); nburke12@fordham.edu (N.L.B.)

**Keywords:** food insecurity, eating disorders, binge eating, body dissatisfaction, body mass index, adolescents, ecological momentary assessment, race/ethnicity, structural inequality, health equity, inclusion

## Abstract

Adolescence is a critical period for the emergence of eating disorders, and food insecurity may be related to eating pathology and weight, as evidenced in adults. However, little is known about food insecurity and eating pathology during this developmental period, and associations between food insecurity and body mass index (BMI) are mixed. Therefore, we examined associations between food insecurity and BMI percentile, self-reported eating-related pathology and binge eating, and subgroup differences by race/ethnicity. In a subset, we examined the relationship between food insecurity and real-world hunger, food craving, and loss-of-control eating using ecological momentary assessment (EMA). Fifty-eight adolescents at two sites (clinical sample, *n* = 38, BMI percentile ≥ 70th; community sample, *n* = 20, all BMI strata) completed self-report questionnaires. Adolescents were 15.2 ± 2.1 years old, 62% female, 50% Black, 34.5% Hispanic, with BMI percentile = 80.5 ± 25.8 (range 4–99). In the full sample, food insecurity was associated with greater BMI (*p* < 0.01), higher shape/weight overvaluation (*p* = 0.04), and greater number of binge eating episodes among those reporting at least one binge episode (*p <* 0.01), with significant relationships for BMI percentile, shape/weight overvaluation, body dissatisfaction, and binge episode frequency among Hispanic adolescents only (each *p* < 0.01). As in adults, food insecurity may be a risk factor for eating pathology, particularly for Hispanic teens.

## 1. Introduction

Adolescence is a period of development that is characterized by increasing rates of obesity and eating disorders. In 2018, the prevalence of obesity among U.S. children and adolescents aged 2–19 was 19.3% [1] and was highest among adolescents (20.6%) compared to school-age (18.4%) and pre-school-aged (13.9%) children [1,2]. Although the prevalence of eating disorders is comparatively low to that of obesity, in a nationally representative survey conducted among U.S. adolescents aged 13–18 from 2001 to 2003, the prevalence of eating disorders was 2.7%, and increased with age (2.4% for ages 13–14, 2.8% for ages 15–16, and 3.0% for ages 17–18) [3]. A large percentage of adolescents also report subthreshold rates of disordered eating [4], which is a known risk factor for the development of full-threshold eating disorders [5,6].

Food insecurity may be a contributing factor to both obesity and eating disorders. Food insecurity is defined as having limited or uncertain access to nutritionally adequate and safe foods or having a limited or uncertain ability to obtain these foods in a socially acceptable manner [7]. Many U.S. adolescent households are categorized as food-insecure, with Black non-Hispanic and Hispanic households experiencing a higher prevalence of food insecurity and very low food security compared to the national average [8]. While food insecurity rates peaked during the Great Recession of 2008, they had been steadily declining over the last decade [9]. However, not only did COVID-19 reverse this trend [10], but the pandemic also disproportionately impacted the health and well-being of racial and ethnic minorities [11], making research in this area critical.

Food insecurity has been associated with obesity in adults. In a population-based online survey of adults, self-reported low and very low food security were associated with having obesity with or without binge eating disorder (BED) [12]. In a review of ten adult studies examining food insecurity and weight outcomes conducted between 2005 and 2012, there was a consistent, positive relationship between food insecurity and obesity [13]; this relationship was particularly strong in women [14]. The adolescent literature regarding food insecurity and BMI/obesity is less consistent. A review of nine studies conducted between 2005 and 2012 reflects mixed findings, with some reporting positive [15,16], null [17,18], and negative [19] relationships between food insecurity and BMI/obesity in children and adolescents [13]. These mixed findings may result from racial or ethnic differences in the relationship between food insecurity and BMI. Bhattacharya and colleagues found a link between self-reported food insecurity and obesity among White and Hispanic, but not African American, children [20]. In adults, food insecurity predicted overweight/obesity in White and Hispanic women, but not Black women, nor White, Black, or Hispanic men [21]. It is possible that the impact of food insecurity on BMI may be influenced by intersectional effects of sex, race, and ethnicity, highlighting the importance of examining potential demographic moderators.

In adults, food insecurity has also been associated with clinically significant eating disorder pathology including dietary restraint, objective and subjective binge episodes (OBE and SBE, respectively), overeating, night eating, and BED [22]. A similar trend has been observed for compensatory behaviors such as vomiting, laxative/diuretic use, skipping meals, and exercise [22]. Objective studies of eating behavior in adults support an association between food insecurity and greater measured food intake. After consuming a 5-day weight-maintaining diet on a clinical inpatient research unit, participants classified as food secure and food insecure were given free access for 23.5 h to a vending machine stocked with palatable food items rated by the Food Preference Questionnaire (FPQ). In this standardized vending machine paradigm, food insecurity was associated with greater food intake and greater kilocalories from fat and carbohydrates [23].

Similar patterns have been observed in adolescents, although there are fewer studies to date. In a population-based study of adolescents from urban neighborhoods, food insecurity was related to fasting, eating very little food, meal skipping, laxative and diuretic use, but not binge eating or vomiting [24]. Another study found that self-reported food insecurity in adolescence predicted binge eating 5 years later, but only in adolescents from low socioeconomic backgrounds [25]. Food insecurity has also been linked to body dissatisfaction in adolescents, an established risk factor for eating pathology [5,26,27]. In this study, across all BMI groups, children with food insecurity had a significantly higher odds of body dissatisfaction than food secure children [28]. This relationship was particularly strong in those with normal weight (compared to children with underweight, overweight, or obesity). Similarly, across racial and ethnic groups, those who were food insecure had higher odds of body dissatisfaction than those who were food secure, especially among African American children [28]. Finally, in a qualitative study of low-income, food-insecure and food-secure households, themes of secretive eating, food hiding, night eating, and binge eating emerged in focus groups for food-insecure, but not food-secure, households [29]. Because eating disorders had historically been thought to only impact “skinny, white affluent women”, research on eating disorders has been primarily conducted in these individuals, leaving a large gap in the literature as to how eating disorders may emerge in racial and ethnic minorities [30]. Considering the disproportionate impact of food insecurity on racial and ethnic minority communities [31], additional studies are necessary to help us better understand how disordered eating emerges in individuals previously excluded from research, including investigation of the role of food insecurity.

Notably, obesity and disordered eating, especially binge eating, have shared risk factors [32] and are frequently co-morbid [33]. One hypothesis regarding the paradoxical relationship between food insecurity and obesity and binge eating is related to the administration of food assistance programs such as Supplemental Nutrition Assistance Program (SNAP, formally called food stamps; benefits are provided to individuals with lower socioeconomic status for the purpose of assisting with purchase of foods and beverages) in the United States [34]. The distribution process of SNAP benefits and other governmental assistance programs is cyclical in nature, possibly fostering a pattern in which overeating occurs in the beginning of the month when money and food stamps are available, followed by a period of involuntary restriction or limited access to food at the end of the month when resources are scarce, further perpetuating overeating when food stamps become available again [35]. Existing models of binge eating, such as restraint theory and the CBT model [36], posit that restriction plays a contributing role in maintaining binge eating behavior by increasing hunger and perceived deprivation. Thus, it is possible that individuals with food insecurity are more susceptible to binge eating by virtue of greater levels of hunger, food cravings, and/or greater variability in hunger/food cravings over time, resulting from inconsistent, unreliable access to food.

Ecological momentary assessment (EMA) provides an ideal method to begin examining this hypothesis, by providing repeated ratings of hunger and cravings in the real world. Many of the studies that have examined food insecurity in the context of eating pathology rely on retrospective self-report measures of eating disordered behaviors and cognitions, which may introduce subjective recall bias. The use of EMA allows real-time examination of affect and behavior with the aid of an electronic device. In adolescents, EMA has successfully been used to study eating behavior generally, and loss-of-control eating, specifically, a key feature of binge eating behaviors [37]. Several studies have used EMA to examine how negative affect [38], cognitions [38], and interpersonal factors [39] relate to loss-of-control eating. To date, no studies have examined the relationship between food insecurity and real-world experiences of hunger, craving, or loss-of-control eating, and doing so has the potential to extend existing findings from self-report studies.

Adolescence is a critical period for the emergence of eating disorders, and food insecurity may be related to eating pathology and weight, as evidenced in adults. There is also data to suggest that food insecurity and eating disorder pathology may be associated in adolescence; however, few adolescent studies have examined differences by racial/ethnic group; most studies only use self-report methodology; and the relationship with BMI is unclear. While previous studies have examined these relationships in secondary analyses, we aimed to do so as a part of our primary aim, which is a strength of the study. We therefore examined associations between food insecurity and: self-reported eating-related pathology, binge eating, and BMI percentile, as well as subgroup differences by race/ethnicity. Based on existing findings, we hypothesized that food insecurity would predict greater eating pathology, binge eating, and BMI percentile. In exploratory analyses, we also examined subgroup differences by race and ethnicity, in light of prior findings suggestive that race/ethnicity plays a moderating role. Finally, to build on the existing self-report literature, we examined the relationship between food insecurity and real-world loss-of-control eating, hunger, and food craving (which is also associated with eating pathology) [40], using EMA. We hypothesized that food insecurity would predict greater mean loss-of-control eating and greater variability in hunger and craving, measured in the natural environment.

## 2. Materials and Methods

Participants were drawn from two adolescent samples (one clinical sample, one community sample) enrolled in ecological momentary assessment studies in the New York City metropolitan region. “Clinical sample” denotes a sample of adolescents who were recruited and included based on reporting recurrent episodes of loss of control or binge eating in the month prior to assessment, but they did not receive treatment as part of study participation. Adolescents received a psychoeducation session at the end of the study, and when clinically appropriate, treatment referrals were provided (i.e., in the case of binge eating disorder or subthreshold binge eating disorder). Initial eligibility was determined via a telephone screening, and potentially eligible participants were scheduled for an in-person appointment, during which the adolescent’s height and weight were measured. Participants also completed a series of questionnaires to assess food insecurity, eating pathology, and obtain demographic information. To meet eligibility criteria for the ecological momentary assessment portion of the study, adolescents in the clinical sample had a BMI percentile ≥ 70th and endorsed at least two loss of control eating episodes within the last month. The community sample did not have any BMI percentile restrictions and included adolescents with and without loss-of-control eating.

Adolescents who were eligible for participation in the study were trained on to how to complete EMA ratings on their smartphone, using a web-based application. Smartphones were provided for those who did not have access to one. For 1 week, adolescents used a secure web-based application to report daily information about their stress, negative affect, hunger, food cravings, loss-of-control eating, and other aspects of eating behavior. Participants were asked to record a response when they received a text message, as well as before and after eating. The programmed texts were scheduled to occur randomly within five stratified intervals between 10:00 and 22:00 h. Participants did not receive texts during the school day, so texts occurred three times per day on weekdays, beginning after school, and five times per day on weekends and weekday school holidays.

Body mass index (BMI; kg/m^2^). Height was recorded to the nearest millimeter using a calibrated stadiometer and weight was measured to the nearest 0.1 kg using a calibrated digital scale. Height and weight were converted to BMI-for-age-and-sex percentiles using the Centers for Disease Control and Prevention growth charts [41].

Demographics. Demographic information was obtained via a parent-report questionnaire that included information about the adolescent’s age, race/ethnicity and sex.

Food insecurity. Adolescents completed the self-report version of the Food Security Survey Module for Youth Ages 12 and Older [42,43] to assess food insecurity in the past month. The raw score was the sum of affirmative responses (0—high food security, 1—marginal food security; 2–5—low food security; 6–9—very low food security). Participants were then categorized as not food insecure (0–1) or food insecure (2–9).

Eating disorder pathology. The Eating Disorders Examination Questionnaire Adolescent Version (EDE-A) [44,45] was used to measure eating disorder pathology. We assessed binge episode frequency with specific questions (items 13 and 14 on EDE-Q and items 18 and 19 of the EDE-A): “Over the past four weeks (28 days) … How many times have you eaten what other people would regard as an unusually large amount of food (given the circumstance)? On how many of these times did you have a sense of having lost control over your eating (at the time you were eating)?” The seven-item, three-factor version of the EDE-Q was applied to generate subscales for shape/weight overvaluation, body dissatisfaction, and dietary restraint based on previous research indicating that the abbreviated, seven-item factor version captures eating pathology equivalently across Black and White adolescents, whereas the original version does not [46]. The three-factor structure has been replicated successfully in clinical and non-clinical samples of Latinx adolescents and young adults [47].

EMA Measures. During the 1-week study period, adolescents responded to texts on their smartphone using a web-based EMA data collection platform (Real-Time Assessment In the Natural Environment, ReTAINE) to assess momentary hunger (“How hungry do you feel?”) and craving (“How much do you agree with this statement: I am craving food.”). The responses were recorded using a visual analog scale (VAS) from 0 to 100, with 0 indicating “not at all” and 100 indicating “extremely” for stress and hunger and “very much” for craving. Level of loss-of-control during eating was measured via a composite of three questions assessing the extent to which the adolescent felt out-of-control or could not stop eating, also assessed on a 0–100 VAS scale.

Statistical analyses. ANOVAs were used to test differences in demographic, independent, and dependent variables by site. Variables were examined for normality (skew > 1, kurtosis > 3). If skew > 1 or kurtosis > 3, transformation was used to improve normality. We used a log transformation for positive skew and reflect then log for negative skew. A series of Univariate ANOVAs were used to assess the effect of food insecurity on each dependent variable including binge frequency, EDE-Q subscales (shape/weight overvaluation, body dissatisfaction, restraint), and BMI percentile. In all models, we considered site (clinical versus community) as a covariate. In models predicting eating-related outcomes, we also included BMI percentile as a covariate. We did not include sex as a covariate due to the small sample size. When covariates were non-significant, they were removed from the model. Due to the large number of adolescents who reported zero binge eating episodes, we used a Poisson model to test the relationship between food insecurity and binge eating. A Poisson model includes two results—a count model (Poisson with log link) and a zero-inflation model (binomial with logit link). The zero-inflation model tests if food insecurity is associated with any binge eating (compares 0s versus all other frequencies). The count model tests if food insecurity is associated with frequency of binge episodes, in individuals with more than one episode. Univariate ANOVA was also used to examine the relationship between food insecurity and means and variability in hunger, craving, and LOC eating. In all models, we considered BMI percentile as a covariate. For race and ethnicity subanalyses, we ran the same univariate ANOVAs, this time including, in the first model, ethnicity (Hispanic versus peer), and in the second, Black versus other race, as well as interactions between food insecurity and ethnicity/race.

## 3. Results

### 3.1. Participant Characteristics

Thirty-eight adolescents who participated in research studies from the clinical sample and twenty adolescents from the community sample completed self-report measures. Three adolescents in the clinical sample did not participate in the EMA portion of the study due to not reporting > 2 LOC episodes or having a BMI percentile < 70, but they completed self-report measures of food insecurity and eating pathology, and therefore, were included in analyses. Adolescents were 15.2 ± 2.1 years old (range 11.1–18.9), 62% female, with a mean body mass index (BMI) percentile of 80.5 ± 25.8 (range: 4–99). As expected by study design, adolescents in the clinical sample had a higher average BMI (mean ± SD = 92.5 ± 19.7) than adolescents from the community sample (mean ± SD = 56.2 ± 18.8) (*p* < 0.01). In the total sample, 29 (50%) were Black or African American, 9 (15%) were Caucasian, and 1 (1.7%) was Native American. Eleven individuals (19%) indicated their race as “Other” and four (6.9%) indicated “Mixed” race; four (6.9%) declined to respond. Of the 19 adolescents who indicated Other/Mixed Race or who did not respond, 13 (68.4%) reported their ethnicity as Hispanic (of these 13 individuals, 8 reported their race as “Other”, 1 reported “Mixed” race, and 4 declined to report their race). The community sample included a greater proportion of Black or African American adolescents (65%) compared to the clinical sample (42%) (*p* < 0.01). There were no differences by site in age, proportion of female participants, or proportion of Hispanic participants (Table 1).

Fifty-nine percent of adolescents were not food insecure and 41% were food insecure and this did not differ by site (*p* < 0.11). A marginally greater proportion of Black/African American adolescents were food insecure (55.2%) compared with the proportion of their peers who were food insecure (44.8%) (*p* = 0.06).

### 3.2. Food Insecurity and BMI

**Figure 1:**
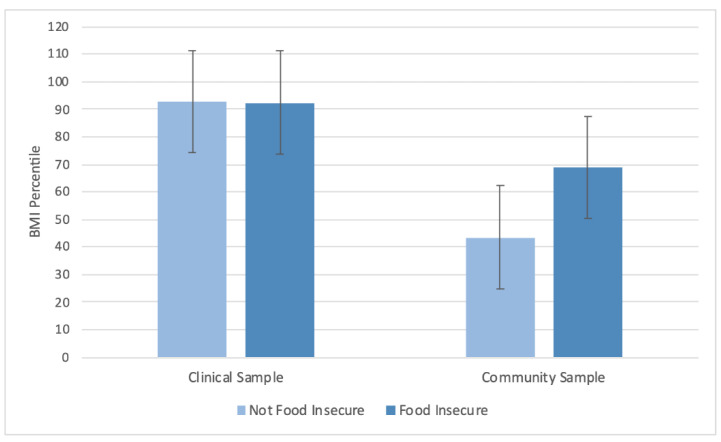
BMI percentile in clinical and community samples. *p*_site_ < 0.01. *p*_food insecurity_ = 0.02. *p*_interaction_ = 0.02. Means ± SD are displayed.

In the model predicting BMI from site and food insecurity, there was a significant main effect of food insecurity on BMI, where food-insecure adolescents had significantly greater BMI percentile (mean ± SD = 80.7 ± 18.6) than those who were not food insecure (mean ± SD = 68.1 ± 21.0) (*p* = 0.02). There was also a significant interaction (*p =* 0.02), such that the relationship between food insecurity and BMI percentile was driven by adolescents from the community sample (Figure 1).

### 3.3. Food Insecurity and Eating Pathology

Controlling for site (*p <* 0.01), there was a significant effect of food insecurity on the overvaluation of shape and weight subscale (*p =* 0.04), but not body dissatisfaction (*p* = 0.11) or dietary restraint (*p* = 0.74) subscales (Table 2). Across sites, food-insecure adolescents (mean ± SD = 2.9 ± 2.0) had greater shape/weight overvaluation compared to adolescents who were not food insecure (mean ± SE = 1.7 ± 2.3) (Table 2).

### 3.4. Objective Binge Episodes

Food insecurity did not predict the presence of binge eating (zero-inflated model) (*p* = 0.6). Among adolescents who reported having at least one binge episode, those with food insecurity reported a greater number of binge episodes (FI mean ± SD = 6.8 ± 5.4, Not FI mean ± SD = 3.3 ± 4.6; *p <* 0.01) (Table 2).

### 3.5. Real-World Hunger, Craving, and LOC Eating

In the models examining food insecurity and loss-of-control, hunger, and craving (mean level and variability) in the natural environment, there were no significant relationships between food insecurity and any EMA variable (Table 3).

### 3.6. Exploratory Analyses

**Figure 2:**
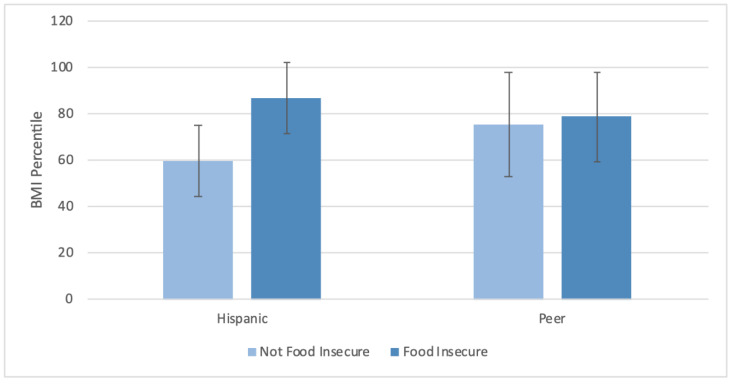
BMI percentile in Hispanic versus peer adolescents. *p*_ethnicity_ = 0.68, *p*_food insecurity_ < 0.01, *p*_interaction_ = 0.04. *p*_food insecurity_ for Hispanic adolescents < 0.01. *p*_food insecurity_ for peer adolescents = 0.63. Mean ± SD are displayed.

The effect of food insecurity on BMI percentile was significant for Hispanic adolescents (*p <* 0.01) but not their peers (*p* = 0.63) (Figure 2).

**Figure 3:**
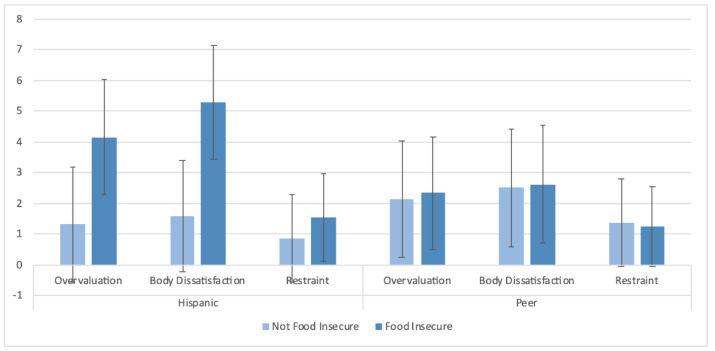
Eating disorder psychopathology in Hispanic versus peer adolescents. In the model including overvaluation/body dissatisfaction, food insecurity, and ethnicity, *p*_interactions_ were significant (for overvaluation, *p_interaction_* = 0.017; for body dissatisfaction, *p*_interaction_ < 0.01). Among Hispanic adolescents: for overvaluation, *p* < 0.01; for body dissatisfaction, *p* < 0.01; for restraint, *p* = 0.88. Among peer adolescents: for overvaluation, *p* = 0.65; for body dissatisfaction, *p* = 0.85; for restraint, *p* = 0.67. Mean ± SD are displayed.

For the EDE-Q subscales, there were significant effects of shape and weight overvaluation (*p* < 0.01) and body dissatisfaction (*p <* 0.01) among Hispanic adolescents, but not their peers (Figure 3).

**Figure 4:**
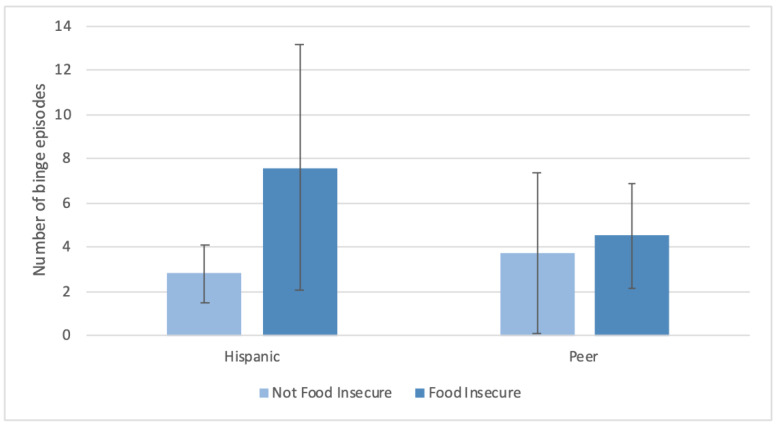
Count model of binge eating episodes among adolescents with at least 1 binge eating episode (*p* = 0.02). Among Hispanic adolescents, *p*_food insecurity_ < 0.001. Among peer adolescents, *p*_food insecurity_ = 0.6. Mean ± SD are displayed.

Among Hispanic adolescents only, those with food insecurity had greater shape/weight overvaluation (mean ± SD = 4.1 ± 1.9 versus 1.3 ± 1.8) and body dissatisfaction (mean ± SD = 5.3 ± 1.8 versus 1.6 ± 1.8) compared to those who were not food insecure. Among those adolescents who reported having at least one binge episode, in the Hispanic subgroup only, food insecurity was associated with a greater number of binge episodes (LVL mean ± SD = 7.6 ± 5.6, Hi-Mar mean ± SD = 2.8 ± 1.3; *p <* 0.001) (Figure 4). There were no significant differences in the analyses comparing Black adolescents and their peers.

## 4. Discussion

In this study, we aimed to build on the existing adolescent studies examining associations among food insecurity, body weight, and eating disorder psychopathology in an a priori investigation of food insecurity. As an exploratory aim, we assessed subgroup differences by race and ethnicity. Our findings suggested that adolescents experiencing food insecurity had a greater average BMI percentile, greater overvaluation of shape and weight, and, among those who had at least one binge episode, greater frequency of episodes. This appeared to be moderated by ethnicity, as when groups were examined separately, significant effects were seen in the Hispanic subgroup only. We did not find a significant relationship between food insecurity and any EMA variables (hunger, craving, and LOC eating).

Regarding eating disorder psychopathology, food insecurity was associated with overvaluation of shape/weight and frequency of binge episodes among those who reported binge eating. Our findings are consistent with prior studies [25,29], but diverge from a recent report in a population-based sample [24] in which no effect was seen. Notably, most prior studies examined whether food insecurity was associated with *any* binge eating, whereas the present results extend the literature by suggesting the possibility that food insecurity increases frequency of episodes in those who have binge eating, but is not associated with whether an adolescent experiences binge eating at all. As with most behavioral and psychiatric disorders, it is possible that the exposure, in this case, to food insecurity, increases risk in a vulnerable subset, but not all teens. The present study is also among the first to examine the relationship between food insecurity and overvaluation of shape/weight and body dissatisfaction—key cognitive symptoms of eating disorders—in adolescents. Consistent with a recent finding in a large sample of middle-school children [48], our results suggested that food insecurity is associated with increases in both symptoms. Further, research suggests food insecurity is related to higher weight status [19,48]. It is plausible that the relationship between food insecurity and these symptoms is moderated by internalized weight stigma [49] or weight-related victimization [50], which are unfortunate, but common, experiences for individuals with overweight or obesity. The absence of significant findings with respect to dietary restraint differs from two adolescent studies [22,24] that showed higher rates of fasting, eating very little, and skipping meals (as well as diuretic and laxative use) in food-insecure teens. However, restrictive eating behavior can be internally or externally driven, and it is possible that adolescents in food-insecure households may not necessarily be attempting to reduce their intake to influence their shape or weight; rather, their restriction may be due to a lack of access to food. Thus, further research could explore this difference more by examining internally versus externally imposed restriction in food-secure and food-insecure households.

The observation that adolescents with food insecurity had a greater average BMI percentile is consistent with prior adult studies [13,14], but differs from the mixed findings in children and adolescents [15,16,17,18,19]. It is possible that mixed findings in adolescents may result from differences in the composition of samples across studies. In our sample, the relationship between food insecurity and BMI was found only among adolescents in the community sample and Hispanic adolescents. Several prior studies that examined subgroup differences similarly found stronger effects in Hispanic (and White) youth compared to African American youth [20]. Additionally, our sample examined adolescents only, whereas several studies showing no association included children under the age of 10 [16,17]. While it is unclear whether age moderates the impact of food insecurity on weight, it is possible that the effect of food insecurity on weight is more distal, such that food insecurity poses a risk for obesity in the future, even if it is not an imminent consequence. Conversely, it is possible that past food insecurity may have lasting effects on eating and weight outcomes, even if an individual is no longer presently experiencing food insecurity, potentially obscuring the relationship between food insecurity and weight. The effect of food insecurity on eating and weight outcomes may also depend on the duration of time an individual experiences food insecurity, and in the present analysis, only past-month food insecurity was assessed. To improve our understanding of the direction and course of this relationship over time, future, longitudinal research with repeated assessment of both food insecurity and weight may better elucidate this relationship.

The results of our exploratory analyses suggest that the effect of food insecurity differed in Hispanic versus peer adolescents, where associations between food insecurity and nearly all eating and weight outcomes were seen in Hispanic adolescents only. Among Hispanic adolescents, food insecurity was associated with greater BMI percentile, overvaluation of shape/weight, body dissatisfaction, and binge episode frequency. In contrast, there did not appear to be racial differences in the effect of food insecurity on eating and weight outcomes among Black versus peer adolescents (in contrast to Altman and colleagues) [28]. Notably, given the small total sample of 58 adolescents, subgroup analyses had low numbers of participants per group, and we were only able to compare adolescents who identified as Hispanic versus peer, or Black versus peer (meaning the comparison groups were heterogeneous). Nevertheless, this finding is consistent with previous studies in the adult literature, which have found links between self-reported food insecurity and obesity among Hispanic (and White) children and adults [20] and suggests that Hispanic adolescents living in food-insecure households may be disproportionately exposed to factors that lead to obesity and eating pathology.

The labeling of groups by race and ethnicity has the potential to obscure the social and structural factors that influence health outcomes. The differences observed in this study across racial and ethnic lines do not reflect biological or “race-based” factors that lead to worse outcomes. Race is a political and social construct [51,52]. It is critical that future research consider the historical, political, and socially constructed circumstances that have led to the systemic oppression of BIPOC populations (Black, Indigenous, and other People of Color) resulting in poorer health outcomes. As highlighted by Buchanan and colleagues, this requires a system-centered approach that is more inclusive in the conduct, reporting, review, and dissemination of research [51]. In this study, the differences observed in Hispanic youth underscore the need to explore how additional societal-level factors, such as access to social services, or neighborhood or school-level resources, and/or family-level factors such as citizenship status, acculturation, or experience of shame/stigma due to food insecurity, moderate the impact of food insecurity on eating-disorder and weight outcomes and potentially explain subgroup differences. With disproportionate rates of food insecurity reported among Hispanic and Black non-Hispanic households compared to the national average [8], identifying mechanisms of ethnic differences may reveal targets for interventions that can be used to support high-risk youth and combat food insecurity and its downstream effects on adolescent physical and mental health outcomes.

The main limitation of the present study is the small sample size and, resulting from that, limited power. Despite the small sample size, significant relationships were still observed. The effect sizes also provide an estimate of the strength of this relationship. Replication of existing findings in studies with higher power is an important future direction. Additionally, study participants were drawn from a convenience sample in the New York City metropolitan area and the resulting demographic breakdown is not representative of the general U.S. population. The use of a convenience sample in our study introduces the possibility of sampling error, and our findings may not be generalizable to other U.S. regions. Future research should include a larger, more representative sample to reflect the national population, as food insecurity is a problem that is not limited to a particular regional subgroup. Of note, while the sample is not reflective of the national population, the oversampling of Black and Hispanic participants is consistent with the disproportionate number of individuals in these groups experiencing food insecurity.

The study is also limited by the selection of categories for race and ethnicity. Race was reported as one of six categories (Black or African American, Caucasian, Asian, Native American, Mixed, and Other) and ethnicity as either Hispanic or non-Hispanic, reflecting NIH guidelines at the start of the study (https://grants.nih.gov/grants/guide/notice-files/NOT-OD-15-089.html, last accessed 26 August 2021). However, the reporting of only one ethnicity in this way fails to capture within-group differences. Participants were not asked their country of origin or nationality and it is possible that the relationships between food insecurity and eating pathology may differ among subgroups. Future research on the effects of food insecurity on adolescent physical and mental health should be more inclusive to reflect the spectrum of identities held by individuals that intersect across racial, ethnic, gender, and social lines [51].

With respect to the EMA findings, a novel and unique contribution to the food insecurity literature, we hypothesized that higher level and variability in hunger and food craving in the natural environment was a potential mechanism by which food insecurity promoted higher BMI and binge eating. However, in our sample, food insecurity was unrelated to levels and variability in hunger and food cravings. It is likely that this is due to our single assessment of food insecurity rather than a daily evaluation of food access and resource allocation variability. Specifically, as resource allocation patterns may foster a cyclical pattern of overeating and restricting, they may also explain hunger and craving variability. We did not account for time of the month (and thus, possible resource availability, such as SNAP benefits) in our analyses, but this is an important consideration for future work, as eating behaviors are likely to change with resource fluctuations. In the EMA phase of the study, we aimed to assess hunger and craving randomly throughout the day; however, some participants only answered these questions several times over the entire study period. For these participants, it is likely that sporadic ratings of hunger or craving may not provide a true representation of their day-to-day experience.

## 5. Conclusions

Despite study limitations, the observed associations found between food insecurity and BMI and eating disorder pathology are generally consistent with a new and growing literature and add evidence to suggest that food insecurity may be a risk factor for excess weight gain and eating disorder pathology, particularly in Hispanic adolescents. While we did not find evidence that food insecurity significantly relates to real-world hunger and craving, this study is also the first to use ecological momentary assessment to capture real-world experiences that may inform mechanisms by which food insecurity affects eating and weight outcomes. Future research using ecological momentary assessment or calendar assessment is warranted to assess periods and patterns of food access for individuals with food insecurity. Assessing food insecurity as a variable construct would allow for more nuanced examination of relations between food insecurity and hunger and craving. In addition, assessment of biological mechanisms of hunger such as ghrelin and leptin hormones may further elucidate the relationship between food insecurity, hunger, and craving, eating disorders, and weight outcomes. Additional longitudinal studies will also help to inform the temporal relationship between exposure to food insecurity and its impact on eating behaviors, eating disorder symptoms, and weight outcomes. Experimental designs observing eating behavior in the laboratory may also help elucidate mechanisms underlying the relationship between food insecurity and disordered eating.

## Figures and Tables

**Table 1:** Participant characteristics.

	Total	Clinical		Community		*p*_site_	*p*_food_	*p*_int_
		Not FI	FI	Not FI	FI			
*N* (%)	58	25 (66)	13 (34)	9 (45)	11 (55)	-	-	0.17
Age (years, mean ± SD)	15.2 ± 2.1	15.6 ± 2.1	15.3 ± 2.1	15.1 ± 2.1	14.1 ± 2.1	0.14	0.30	0.55
Sex (*n*, %, Female)	36 (62.1)	16 (64)	10 (76.9)	4 (44.4)	6 (54.5)	0.25	0.59	-
Race (*n*, %, Black)	29 (50)	8 (32)	8 (61.5)	5 (55.5)	8 (72.7)	<0.01	0.06	-
Ethnicity (*n*, %, Hispanic)	20 (34.5)	9 (36)	5 (38.5)	4 (44.4)	2 (18.1)	0.46	0.32	-

Abbreviations: Not FI = not food insecure, FI = food insecure; *p_site_* _=_ *p* value for site (community versus clinical) *p*_food_ = *p* value for food insecurity (not food secure versus food secure); *p*_int_ = *p* value for the interaction term (site * food insecurity).

**Table 2:** Eating disorder pathology.

	Clinical		Community		Site		Food Insecurity		Interaction	
	Not FI (Mean ± SD)	FI (Mean ± SD)	Not FI (Mean ± SD)	FI (Mean ± SD)	*p*	η_p_^2^	*p*	η_p_^2^	*p*	η_p_^2^
Overvaluation	2.9 ± 2.0	3.8 ± 2.2	0.5 ± 2.1	1.9 ± 2.0	<0.01	0.24	0.04	0.08	0.68	<0.01
Body Dissatisfaction	3.1 ± 2.9	4.2 ± 2.0	1.5 ± 2.1	2.1 ± 2.0	<0.01	0.13	0.11	0.05	0.66	<0.01
Dietary Restraint	1.8 ± 1.3	1.6 ± 1.3	0.4 ± 1.3	0.8 ± 1.3	<0.01	0.15	0.74	<0.01	0.34	0.02
Objective Binge episodes	3.3 ± 3.3	4.7 ± 3.3	0	9.0 ± 3.3	0.06	-	<0.01	-	-	-

^1^ Abbreviations: Not FI = not food insecure, FI = food insecure.

**Table 3:** Ecological momentary assessment of hunger, craving, and LOC eating.

	Not Food Insecure (Mean ± SD)	Food Insecure (Mean ± SD)	*p*	η_p_^2^
Mean Hunger	22.9 ± 19.2	31.6 ± 19.2	0.12	0.51
Hunger Variability	20.1 ± 11.1	20.4 ± 11.1	0.93	<0.01
Mean Craving	22.4 ± 21.2	30.3 ± 21.2	0.20	0.04
Craving Variability	16.8 ± 10.9	20.8 ± 10.9	0.21	0.04
Mean LOC	21.2 ± 15.2	25.2 ± 15.2	0.44	0.02
LOC Variability	16.4 ± 9.2	16.8 ± 9.2	0.90	<0.01

## Data Availability

Deidentified data may be available upon reasonable request from the investigators by emailing the corresponding author.
